# Ethnic Differences in DNA Methyltransferases Expression in Patients with Systemic Lupus Erythematosus

**DOI:** 10.1007/s10875-012-9803-z

**Published:** 2012-10-09

**Authors:** Kenneth L. Wiley, Edward Treadwell, Kayihura Manigaba, Beverly Word, Beverly D. Lyn-Cook

**Affiliations:** 1 FDA-National Center for Toxicological Research, 3900 NCTR Rd, Jefferson, AR 72079 USA; 2 East Carolina Brody School of Medicine, Greenville, NC USA

**Keywords:** Lupus, DNA methyltransferases, DHEA, DNMT3A, DNMT3B, DNMT1

## Abstract

**Purpose:**

Systemic lupus erythematous (SLE) is a systemic autoimmune inflammatory disease with both genetic and epigenetic etiologies. Evidence suggests that deregulation of specific genes through epigenetic mechanisms may be a contributing factor to SLE pathology. There is increasing evidence that DNA methyltransferase activity may be involved. This study demonstrated modulation in expression of DNA methyltransferases (DNMTs) according to ethnicity in patients diagnosed with SLE. Furthermore, differential expression in one of the DNMTs was found in a subset of lupus patients on dehydroepiandrosterone (DHEA) therapy.

**Methods:**

Real-time PCR analyses of DNMT1, DNMT3A and DNMT3B in peripheral blood mononuclear cells from a cohort of African American and European American lupus and non-lupus women were conducted. Also, global DNA methylation was assessed using the MethylFlash^TM^ methylated quantification colorimetric assay.

**Results:**

Significant increase in DNMT3A (*p* < 0.001) was shown in lupus patients when compared to age-matched healthy controls. This increase was associated with a higher SLEDI index. More striking was that expression levels for African American (AA) women were higher than European American women in the lupus populations. A subset of AA women on DHEA therapy showed a significant decrease (*p* < 0.05) in DNMT3A expression in comparison to lupus patients not on the therapy. DHEA is an androgenic steroid found in low levels in the serum of lupus patients. Supplementation of this hormone has been shown to be beneficial to some lupus patients. DHEA was not shown to effect DNMT1 or DNMT3B expression. Increased expression was also noted in DNMT3B (*p* < 0.05) in lupus patients compared to age-matched healthy controls. However, no significant difference was noted in DNMT1 (*p* = 0.2148) expression between lupus patients and healthy controls. Although increases were detected in de novo methyltransferases, a global decrease (*p* < 0.001) in 5-methycytosine was observed in lupus patients when compared to age-matched healthy controls.

**Conclusion:**

These findings suggest that epigenetic changes may play a critical role in the manifestations of the disease observed among ethnic groups, particularly African American women who often have a higher incidence of lupus. DHEA therapy effects on DNMT3A expression in AA women warrant further investigation in a larger population.

## Introduction

Systemic Lupus Erythematosus (SLE) is an autoimmune disease that has multifaceted effects on its sufferers. Women, in general, account for a significant majority of SLE patients [1]. Furthermore, African-American and Hispanic women have a significantly increased risk of developing SLE as compared to their European American counterparts [2, 3]. However, very few studies have been conducted in these populations. Until the recent FDA approval of Benlysta (belimumab), there had not been an FDA approved drug therapy for lupus since 1955 and physicians were forced to use off-label medication to treat the symptoms of SLE [4–8]. However, the use of these off-label drugs lead to the development of toxic side effects that increased the complication in treating individuals with SLE [9–11]. In addition, Benlysta has been approved to treat only symptoms that are primarily involved with musculoskeletal and mucoscutaneous complications with lupus [12]. Furthermore, limited data available indicate that Benlysta does not provide a therapeutic benefit among African Americans, a population with a high rate of susceptibility to SLE [12].

The difficulties in designing an effective pharmacological therapy for SLE are due in part to the complexities of its pathophysiology. Studies are being conducted to determine why women are more susceptible than men to developing SLE. There has been evidence that both estrogen and prolactin can induce lupus in mice, leading researchers to suggest that there is a link between estrogen, prolactin and SLE [13]. Low serum levels of DHEA have also been observed in some patients with lupus. Studies have shown that DHEA treatment improves the patients’s overall quality of life [14]. Recently, there has been increased evidence of an epigenomic component in the pathology of SLE [15, 16]. For example, studies have shown that using 5-azacytidine (a DNA methyltransferase inhibitor) on CD4^+^ T cells can lead to autoreactivity [17]. Studies have also shown that DNA demethylation can lead to increased activation of CD40LG, which leads to increased expression and B cell activation. Zhou et al. [18] showed that this increased expression of CD40LG in women with SLE was due to reactivation of the receptor’s gene on the inactive X chromosome. These studies are among those that show a link to epigenetic regulation, specifically DNA methylation, and autoimmunity. DNA methylation is important in development and maintaining homeostasis of an organism. From cellular reprogramming to the development of the immune system, methylation of an organism’s genome is crucial for survival [19, 20]. Methylation of DNA occurs in two forms: de novo and maintenance methylation, and enzymes that catalyze these reactions are DNA methyltransferases (DNMTs) [21]. Of the six known DMNTs: DMNT1, DNMT2 (TRMDT1), DMNT3A, DMNT3B and DMNT3L, only three are active methyltransferases. There is also evidence of a link between DNMT3B function and disorders such as immunological disorders, centromeric instability, and facial abnormalities (ICF). Mainly, evidence associated with these disorders are due to germline mutations in DNMT 3B [22]. There is data to support the fact that patients who suffer from SLE have decreased levels of DMNT3B [15, 23]. Low levels of DMNTs can lead to hypomethylation, which leads to reactivation of otherwise silent genes. Global methylation analyses have been conducted with SLE patients and have shown that global hypomethylation occurs in patients with SLE [19, 24]. Unfortunately, most of these studies were not conducted in African American women. There is a lack of data on epigenetic changes in African American women for comparison observation seen in other populations. African Americans women are diagnosed with SLE at a higher rate than other populations in the United States. With a scarcity of data regarding a population that makes up a significant portion of patients diagnosed with SLE, and growing evidence suggesting that DNA methylation is linked to the pathogenesis of SLE, this investigation was conducted to determine if ethnic differences exist in epigenetic changes. This study examined the expression level of DNMTs 1, 3A and 3B in a multi ethnic population that has been diagnosed with SLE. The results from this study can contribute to research assessing whether targeting specific methyltransferases would be beneficial for patients with SLE in certain populations.

## Materials and Methods

### Subjects

All subjects in this study gave their informed written consent to be included in the study. The patients that participated in this study were part of the LUPUS study at the Brody School of Medicine at East Carolina University. The cohort for this study consisted of a total of 224 participants. The participants representing this cohort were further categorized in the following manner: 114 individuals diagnosed as having SLE based on SLE disease activity index (SLEDAI) score and by anti-dsDNA antibody analysis, and 110 controls; all were age, sex and ethnicity matched. This project was granted IRB approval from both The Department of Health and Human Services Food and Drug Administration and from East Carolina University.

### Blood Collection and Isolation of PBMC

For our analysis, blood samples were collected by venipuncture of the antecubital vein between 9:00AM–12:00PM. In order to maintain a similar circadian pattern between our participants with SLE and their matched control participants, collections were conducted at the same time of day and the same day of the week. Peripheral blood cells were isolated from whole blood using a PAXgene RNA Blood kit (QIAGEN, Valencia, CA). Blood samples were immediately placed in PAXgene kit at East Carolina University in Greenville, NC and shipped immediately in dry ice to the National Center for Toxicological Research for analysis.

### RNA Isolation and Quantitative Real Time PCR

RNA was extracted from peripheral blood mononuclear cells (PBMC) by using a PAXgene RNA kit (QIAGEN, Valencia, CA). After extraction, all RNA samples were tested for their integrity and concentration using a Bio-Rad Experion Automated Electrophoresis System (BIO-RAD, Hercules, CA). cDNA were synthesized from total RNA extractions using a Clontech Advantage® RT-for-PCR Kit (Clontech, Mountain View, CA). DNMT1, DNMT3A and DNMT3B expression analysis was conducted using a Bio-Rad IQ5 quantitative Real Time Polymerase Chain Reaction Detection System (BIO-RAD, Hercules, CA). As a comparison, GAPDH was selected as our housekeeping gene and was used as an endogenous control. qRT-PCR conditions were as follows: 50 °C for 2 min, 95 °C for 10 min (95 °C for 10 s, 56 °C for 45 s, 72 °C for 30 s) for 30 cycles. Relative quantitations of DNMT1, DNMT3A, and DNMT3B mRNA expressions were normalized to GADPH and fold changes were calculated using a 2^−ΛΛCT^ method. Primers utilized for both the methytransferases and GAPDH are listed below:

**Table:** 

Primers	Sense	Antisense
DNMT1	GTGGGGGACTGTGTCTCTGT	TGAAAGCTGCATGTCCTCAC
DNMT3A	CCGGAACATTGAGGACATCT	CAGCAGATGGTGCAGTAGGA
DNMT3B	AGGGTGCGAGCTGGCAAGAC	AATTTCCTACTGCCTGCACGACG
GADPH	GAAGATGGTGATGGGATTTC	GAAGGTGAAGGTCGGAGTC

### Global DNA Methylation Quantification

Global methylation was conducted using a MethylFlash Methylated DNA Quantification kit (Epigentek Inc., Farmingdale, NY). The instructions were followed as outlined in the kit. Briefly, DNA (100 ng) was bound to strip wells that were specifically treated to have a high DNA affinity. The methylated fraction of DNA is detected using capture and detection antibodies and then quantified colorimetrically by reading the absorbance in a microplate spectrophotometer. The amount of methylated DNA is proportional to the OD intensity. The absolute amount of methylated DNA was quantified from a standard curve. The slope of the standard curve using linear regression was done followed by the calculation for the percentage of methylated DNA (5-mC) in total DNA using the below formula.

$$ 5 - {\mathrm{mC}}\left( {\mathrm{ng}} \right) = \frac{{{\mathrm{Sample}}\,{\mathrm{OD}} - {\mathrm{ME}3}\,{\mathrm{OD}}}}{{{\mathrm{Slope}} \times 2}} $$

$$ 5{ - \mathrm{mC}}\left( {\mathrm{ng}} \right) = \frac{{5{\mathrm{mC}}\,{\mathrm{Amount}}\left( {\mathrm{ng}} \right)}}{\mathrm{S}} \times 100 $$

**S** is the amount of input sample DNA in ng. **ME3** is the negative control.

### Statistical Analysis

For this study, a comparison analysis was conducted for each of the aforementioned methyltransferases. The comparisons included participants with SLE to our control population and African Americans with SLE compared to European Americans with SLE. A two-tailed Mann Whitney t-test was used to determine if significant differences existed. For comparison of multiple groups an ANOVA test was used. To compare values, P-values of <0.05 were considered significant. All analyses were performed using Prism.

## Results

**Fig. 1:**
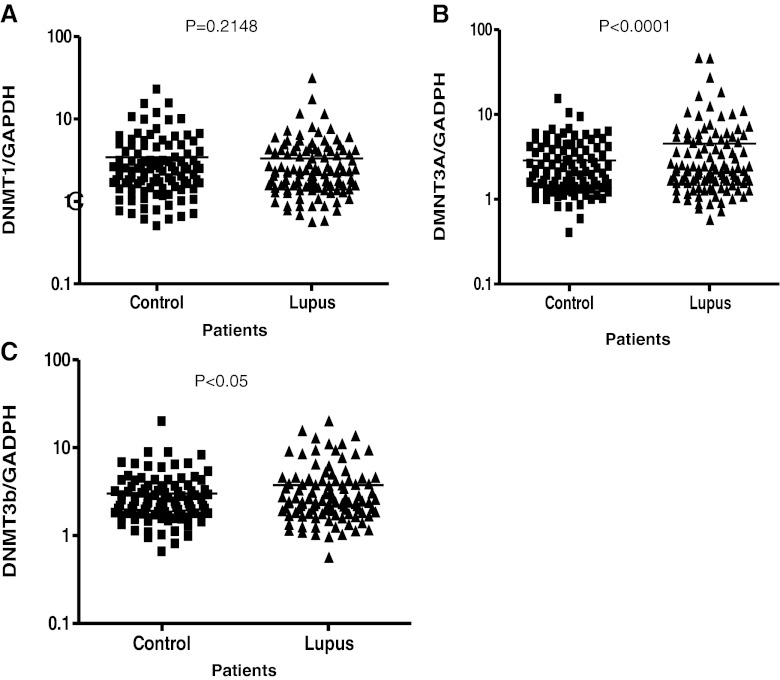
**a** Fold change (2^−ΛΛCT^) comparison of DNMT1 expression for both African American and European American women with SLE vs. aged, ethnicity and sex matched Controls. GAPDH was used as the housekeeping gene for comparison. **b** Fold change (2^−ΛΛCT^) comparison of DNMT3A expression for both African American and European American women with SLE vs. aged, ethnicity and sex matched Controls. GAPDH was used as the housekeeping gene for comparison. The *p* < 0.0001 which indicated there was a significant differences between the two populations. **c** Fold change (2^−ΛΛCT^) comparison of DNMT3B expression for both African American and European American women with SLE vs. aged, ethnicity and sex matched Controls. GAPDH was used as the housekeeping gene for comparison. The *p* < 0.05 which indicated there was significant difference between the two populations

**Fig. 2:**
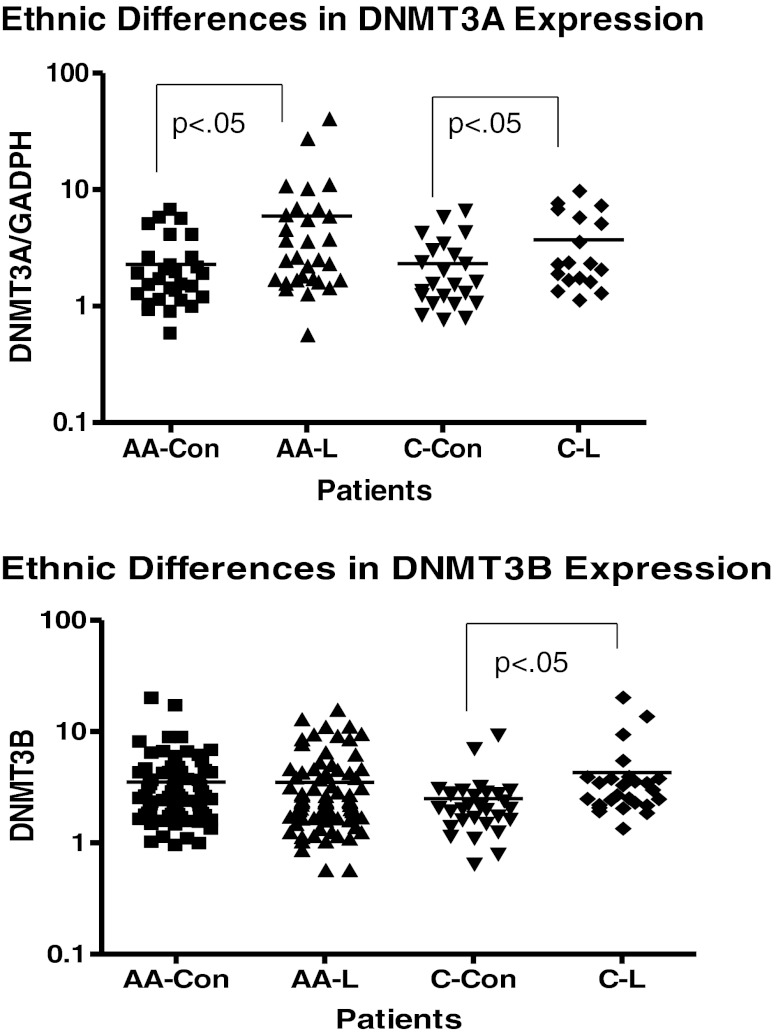
**a** Ethnic Differences in Expression of DNMT3A. AA-Con represents African American Women Controls, AA-L represents African American Women with SLE, C-Con represents European American Women Controls and C-L represents European American Women with SLE. **b** Ethnic Differences in Expression of DNMT3B. AA-Con represents African American Women Controls, AA-L represents African American Women with SLE, C-Con represents European American Women Controls and C-L represents European American Women with SLE

**Fig. 3:**
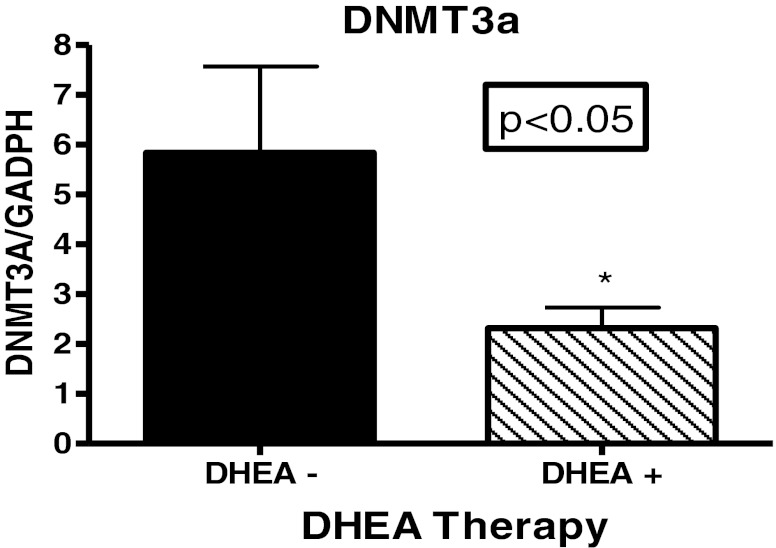
Fold change (2^−ΛΛCT^) comparison of DNMT3A expression in African American women with SLE vs. DHEA therapy. GAPDH was used as the housekeeping gene for comparison. The *p* < 0.05 which indicated there was significant difference between those lupus patients on DHEA and those patients without DHEA therapy

**Fig. 4:**
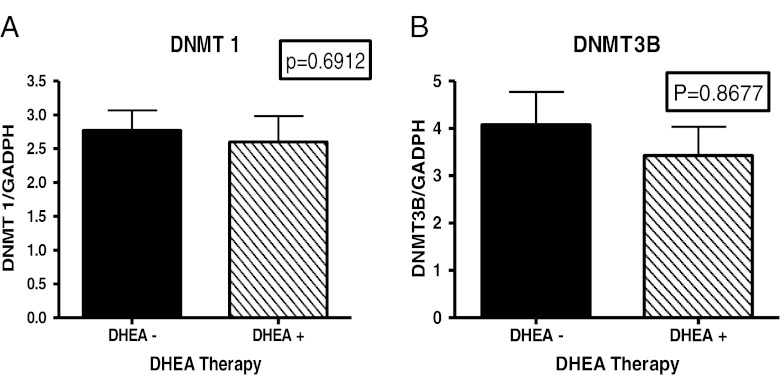
Fold change (2^−ΛΛCT^) comparison of DNMT1 and DNMT3A expression in African American women with SLE vs. DHEA therapy. GAPDH was used as the housekeeping gene for comparison. There was no significant difference between those lupus patients on DHEA and those patients without DHEA therapy

**Fig. 5:**
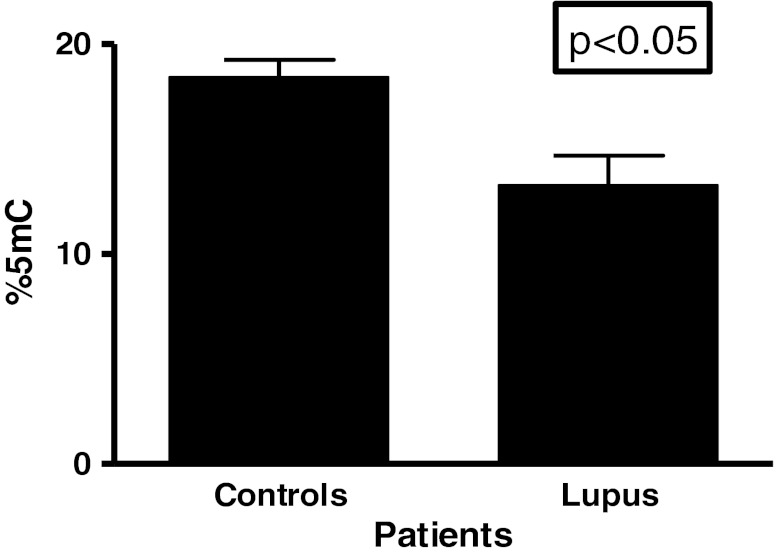
Global 5-methylcytosine levels in healthy controls and lupus patients. A significant (*p* < 0.05) decrease was noted in lupus patients

Figure 1a shows results from comparisons of expression of DNMT1. The results from this study indicated that there were no significant difference in the mRNA expression levels of DNMT1 among patients with SLE compared to aged-matched controls. There was evidence of inter-individual variation but the means of the two groups were not significantly different (*P* = 0.2148). DNMT1 is predominantly, but not exclusively, involved with maintenance methylation [25, 26]. Therefore, based on the results from our DNMT1 expression analysis, maintenance methylation may not play an active role in the disease progression of SLE in these populations. However, results from analysis of methyltransferases that are involved with de novo methylation demonstrated that their expression levels differ. Figure 1b shows the results from comparison of expression levels of DNMT3A. With a p-value less than 0.0001, analysis indicates that expression levels of DNMT3A was significantly higher in SLE population compared to controls. In addition, Fig. 1c shows that DNMT3B expression levels are also significantly higher in our SLE population compared to aged, sex and ethnicity matched controls (*p* < 0.05). Based on our initial results, we then proceeded to look at expression levels of methyltransferases according to ethnicity. Figure 2a indicates that both African American and European American women, who are diagnosed with SLE, have a significantly higher expression level (*p* < 0.05) of DNMT3A compared to their respective control counterparts. What is most surprising is the difference in DNMT3A expression between ethnicity. It was found that African American women with SLE had a significantly higher expression of DNMT3A compared to European American women with SLE. Figure 2b shows ethnic differences in DNMT3B. There was no difference in expression of DNMT3B in African American lupus women when compared to controls (*P* = 0.9708); however, there was a significant difference in European American lupus women when compared to their controls (*P* < 0.05). There were no difference between African American and European American lupus women (*P* = 0.3064). In comparing the mean expression level of DNMT3A in patients with a higher SLEDAI (>4), expression was higher than those with SLEDAI (<4) (*P* = 0.0120) (Table I). Furthermore, examining those lupus patients on DHEA therapy, it was found that African American women had a significant decrease in expression of DNMT3A (Fig. 3). However, no effect was found on expression of DNMT1 and DNMT3B (Fig. 4). These results indicate not only evidence of de novo methylation activity involvement in the pathogenesis of SLE, but also that African American women with SLE had a higher expression of DNMT3A compared to European women with SLE. Although DNMTs 3A and 3B expression levels were increased, this study also confirmed other studies in showing a global decrease in 5-methycytosine in lupus patients compared to age-matched healthy controls (Fig. 5).

**Table I:** Comparison of DNMT3A expression with disease status

	SLEDAI (>4)	SLEDAI (<4)
DNMT3A	5.717 ± 1.24	2.527 ± 0.257 (*P* = 0.0120)

## Discussion

Recently, emerging evidence is revealing the role of epigenetic dysregulation in the pathogenesis of systemic lupus erythematosus [27–31]. Epigenetics refers to heritable modifications that are known to modulate gene expression without changes to DNA sequences. Epigenetic activation and inactivation of genes involve several components, DNA methyltransferases, histone deacetylases, histone acetylases, methyl binding proteins, DNA methylases and in some cases, microRNA [32–36]. Although most studies, including this study, have shown overall global hypomethylation, recent studies have indicated regional hypermethylation in a recent case–control study using high-throughput methylation arrays. This study revealed the methylation status of over 27,000 CpG sites in the promoter regions of about 15,000 genes of which 236 sites were hypomethylated and 105 sites were hypermethylation in female lupus patients [37]. Epigenetic regulation by CpG methylation at specific sites in T cells controls the differentiation of T helper cells [38, 39]. Recently, a study showed that the Foxp3 gene, which acts as the master regulator of the development of regulatory T-cells, expression level is decreased in lupus patients and further suggested promoter DNA methylation as a possible mechanism [40].

This study investigated modulation of DNA methyltransferases in lupus and non-lupus patients. Although conflicting results exist on the level of DNMTs expressed in lupus patients, this may be due to variability in the disease activity of the population studied. DNMT3a and DNMT3b produce *de novo* methylation. These methyltransferases play an important role in gene silencing by hypermethylation of promoter regions within an organism’s genome. This study indicated expression level differences in methyltransferases primarily involved with de novo methylation among patients who are diagnosed with SLE compared to the control population. However, DNMT1, which is principally involved in maintenance methylation, did not show a significant different expression level between lupus and non-lupus patients. Mixed results have been found in other studies analyzing DNMT1 expression in SLE patients from other populations. For example, in studies conducted among Taiwanese populations with SLE, DNMT1 expression levels were significantly increased compared to their controls [41]. But, in studies conducted using a population with SLE from Shanghai, there was a decrease in DNMT1 expression [19, 24]. In the present investigation, SLE patients have a significantly increased expression level of DNMT3A compared to the control population. In addition, we observed that African American women with SLE have significantly higher expression levels of DNMT 3A compared to European American women with SLE. High levels of de novo methyltransferases correlates with hypermethylation or the silencing of specific genes through promoter hypermethylation.

Our studies have further shown that those patients with a SLEDAI of greater than four (4) had higher expression level of DNMT3A. Although most animal studies and limited human studies have shown increased expression of specific genes in lupus patients, possibly through hypomethylation, more studies are needed to identify those genes which may be inactivated through promoter hypermethylation. A study conducted with subjects in China showed no difference between DNMT3A expression and DNMT3B expression between patients with SLE and their control counterparts [42]. The factors contributing to differences shown in different studies remain unknown. However, studies have shown a correlation between increased methyltransferase expression and lupus patients in the active stage of the disease. This study shows for the first time significant changes in DNMT3A expression in African American women. Although this study showed a contrast with other studies in DNMT expression, it confirmed other data in detecting an overall decrease in global 5-methylcytosine activity in lupus patients, which could lead to hypomethylation of methylated-sensitive genes. Global hypomethylation and increased methyltransferase expression and activity have been shown in other diseases such as cancer [41]. A number of reasons could be suggested for epigenetic differences seen among various populations, such as dietary, occupational exposures, and viral infections [23, 24]. Studies have also shown that early life environmental influences, such as exposure to infectious agents, can lead to modulation of DNMTs expression and autoantibody production later in life [42]. Also, because a number of medications are often given to lupus patients to relieve symptoms, these factors could also contribute to the epigenetic changes. Although studies are underway to correlate patient medications with changes shown in DNMTs and global methylation, previous studies have shown no correlation [41]. However, our study found that a subset of African American lupus patients receiving DHEA therapy showed decreases in DNMT3A expression. Studies have shown low levels of DHEA in the serum of lupus patient and that supplemention of this hormone improved their overall quality of life [42]. The epigenome may be playing a more active role than researchers have believed in the disease progression of SLE and may be a determining factor in active *vs* inactive status of lupus patients. Indeed, several studies have shown a role for DMNTs in autoimmunity. For example, studies have shown that tranforming growth factor beta (TGF-β) and 5-azacytidine (5AzadC) (a DNMT inhibitor) can work to induce increased expression of Foxp3 in human T cells [43]. This implies that DNMT plays a role in human T cell development and function. In addition, interleukin 4 (Il-4) expression, which plays a role in differentiation and function of T-cells, is linked to DNA methyltransferases [44]. There is also evidence that shows interleukin 6 (Il-6), whose secretion can cause SLE like symptoms by down regulating JunB, can effect the nuclear translocation of DNMT1 [45, 46].

The fact that this study shows increased expression levels of DNA methyltransferases related to de novo methylation instead of those related to maintenance methylation may not be by coincidence. However, the fact that there is a specific form of methylation that is more active in lupus patient above others provides more evidence that the epigenome is more then just a simple observer in the disease progress of SLE. Epigenetic targets are currently being explored for drug development for SLE.
